# Outcomes of Surgical and Mechanical Thrombectomy in Massive Saddle Pulmonary Embolism: A National Perspective

**DOI:** 10.7759/cureus.29885

**Published:** 2022-10-03

**Authors:** Nishok Victory Srinivasan, Jorge E Maldonado, Andrew Melek, Faris M Haddad, Achint A Patel

**Affiliations:** 1 General Surgery, California Institute of Behavioral Neurosciences & Psychology, Fairfield, USA; 2 General Surgery, Jackson Memorial Hospital, Miami, USA; 3 Medical School, Xavier University School of Medicine, Oranjestad, ABW; 4 Radiology, Mutah University, Al Karak, JOR; 5 Internal Medicine, HCA Healthcare, University of South Florida Morsani College of Medicine Graduate Medical Education, Brooksville, USA; 6 Internal Medicine, Oak Hill Hospital, Brooksville, USA

**Keywords:** surgical thrombectomy, mechanical thrombectomy, outcomes, predictors, trends, saddle pulmonary embolism

## Abstract

Introduction

Saddle pulmonary embolism (PE) is a type of central PE that involves the bifurcation of the pulmonary arteries. First-line treatment is usually systemic thrombolytics, but surgical and mechanical thrombectomy (ST and MT) are used for patients with contraindications to thrombolytics or right heart strain. This study compares surgical and mechanical thrombectomy trends and outcomes in patients with saddle PE.

Methods

The data was extracted from the National In-Patient Sample (NIS) from 2016-2018 using the International Classification of Diseases-10-Clinical Modification (ICD-10-CM) diagnosis codes. We used the Cochrane-Armitage trend test to analyze the trends of ST and MT and the chi-square test for statistical analyses. A two-tailed p-value of <0.05 was considered statistically significant.

Results

The overall trend of MT in saddle PE rose from 2016 to 2018, while ST remained stable. Around 95% of patients undergoing ST/MT were emergent admissions, with 82.5% occurring in teaching hospitals. Patients of age >65 years and more with comorbidity burdens were more likely to undergo MT over ST. In-hospital mortality after ST was 15.1%, and after MT was 11.1% (p:<0.001). The most common complications after ST were congestive heart failure (CHF) and atrial fibrillation (AF), and after MT were vascular events and CHF.

Conclusion

The use of mechanical thrombectomy has steadily increased during the study period. ST is more common in large/teaching hospitals, weekend admissions, and patients transferred from other facilities. MT is more common in elderly patients with a higher comorbidity burden. Patients who underwent MT had lower mortality, length of hospital stay, and post-procedural complications.

## Introduction

Acute pulmonary embolism (PE), a manifestation of venous thromboembolism, is the third-most common form of cardiovascular death after myocardial infarction and stroke [1]. According to physiological effects, pulmonary emboli may be classified as high-risk (super-massive or massive), intermediate-risk (sub-massive), or low-risk [1]. With the advent of CT pulmonary angiography as the gold standard for diagnosing pulmonary embolism, the exact anatomic locations of PE can also be determined. Based on the level of proximal extension, PE can be anatomically classified as central, lobar, segmental, and sub-segmental [2]. Central pulmonary embolism is diagnosed when thrombi are found in the main trunk of the pulmonary artery and the right or left pulmonary arteries [3]. Saddle PE refers to a specific type of central PE-an embolus lodged in the bifurcation of the main pulmonary artery trunk, often extending into the right and left pulmonary arteries [4,5]. Saddle emboli are more commonly (but not always) high-risk (super-massive or massive) or intermediate-risk (sub-massive) [6].

The conventional treatment of saddle PE is immediate high-dose unfractionated heparin followed by systemic fibrinolysis or thrombectomy [7]. However, major bleeding with systemic fibrinolysis is more common in massive PE: in an overview of five clinical trials, fibrinolysis almost doubled the risk of significant bleeding [8]. As an alternative to fibrinolysis or for patients with contraindications to fibrinolysis, surgical or mechanical thrombectomy can be employed to rapidly reverse PE-related right ventricular failure and cardiogenic shock [7]. A saddle configuration currently does not alter the treatment configuration. However, there is evidence of a higher rate of hemodynamic compromise and complications (cardiac arrest, shock, respiratory failure, mechanical ventilation, length of hospital stays) in saddle PE [6,9], although there is no significant difference in short-term mortality between the saddle and non-saddle PE [6,10].

This study aimed to compare the trends and in-hospital outcomes of surgical and mechanical thrombectomy in treating hospitalized patients with saddle PE. We utilized one of the most extensive population-based datasets to produce national estimates from hospitalized saddle PE patients.

## Materials and methods

Data source

We extracted our study cohort from the National Inpatient Sample (NIS) of the Healthcare Cost and Utilization Project (HCUP), Agency for Healthcare Research and Quality (AHRQ) [11]. NIS is one of the largest all-payer publicly available databases on inpatient discharges from U.S. hospitals maintained by the AHRQ [11]. The NIS approximates a 20% stratified sample of discharges from US community hospitals, excluding rehabilitation and long-term acute care hospitals, and contains more than 7 million hospitalizations annually [11]. With the established survey weights in NIS, this data could be weighted to represent the standardized U.S. population and obtain national estimates with high accuracy [12].

Study population and design

NIS data from 2016-2018 were queried using International Classification of Diseases, 9th Revision, Clinical Modification and International Classification of Diseases, 10th Revision, Clinical Modification (ICD-10-CM) diagnoses codes of I26.02, I26.02 for Saddle PE. Mechanical thrombectomy and surgical thrombectomy were identified by using ICD-10-CM procedural codes. In our final analysis, we only included patients who underwent either surgical or mechanical thrombectomy (ST or MT). Detailed data regarding patient demographics and hospital-level characteristics such as geographical region, size, and teaching status were extracted as supplied by NIS [13]. We estimated comorbidities using Elixhauser comorbidity software [14].

Statistical analysis

To establish the trend, we calculated the proportion of hospitalizations among saddle PE patients who underwent either MT or ST each year and used the Cochrane-Armitage trend test for analysis. We performed descriptive statistics to present the baseline characteristics of saddle PE patients who underwent either MT or ST. We also estimated post-procedural outcomes by thrombectomy type and compared them using the chi-square test. We utilized SAS 9.4 (SAS Institute, Cary, NC) for all analyses and included designated weight values to produce nationally representative estimates [12]. We considered a two-tailed p-value <0.05 as statistically significant.

## Results

Temporal trends of MT and ST

**Figure 1:**
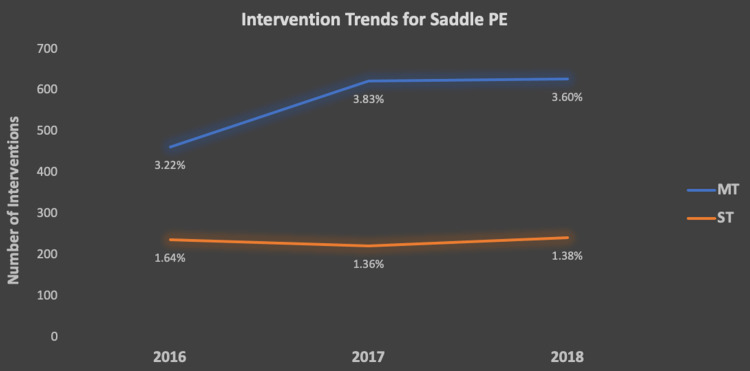
Trends of saddle PE and treatment PE – pulmonary embolism; ST – surgical thrombectomy; MT – mechanical thrombectomy

Between 2016 and 2018, there were ​​47,820 hospitalizations for saddle PE. Of those patients, 1705 underwent MT, and 695 underwent ST. The percentages of each intervention compared to the total admissions are also listed in the figure below (Figure 1). We observed a rise in the use of MT, from 3.22% (n = 460) in 2016 to 3.83% (n = 620) in 2017. Alternatively, ST as an intervention had remained stable over all three years, with no significant differences observed.

Baseline characteristics

The overall population included more males than females (53% vs. 47%) and more white people than other ethnicities (69% vs. 31%). However, there was no significant gender or race difference among the patients undergoing ST vs. MT. A higher proportion of patients in the >65 years age group experienced MT over ST, while the opposite was true for the 18-34- and 35-49-years age groups (p<0.0001). Patients with higher comorbidity scores tended to undergo MT over ST, while those with lower scores underwent ST over MT (p<0.0001). When looking at specific complications, patients with hypertension, uncomplicated diabetes mellitus, chronic pulmonary disease, iron-deficiency anemia, and metastatic cancer were preferentially assigned to the MT group (p<0.05). On the other hand, more patients with obesity, complicated diabetes mellitus, congestive heart failure, valvular heart disease, coagulopathies, and fluid-electrolyte disorders underwent ST over MT (p<0.05). 95% of patients undergoing ST or MT were emergent or urgent admissions, with 82.5% of ST/MT occurring in teaching hospitals. Patients admitted directly from the hospital’s emergency department tended to undergo MT, while those transferred from other facilities had a higher chance of undergoing ST (p <0.0001). Similarly, patients admitted on weekdays had a higher rate of MT, while those admitted on weekends had a higher rate of ST (p = 0.004). Table 1 describes detailed baseline characteristics of saddle PE patients who underwent either MT or ST.

**Table 1:** Baseline characteristics MT – mechanical thrombectomy; ST – surgical thrombectomy; HMO – health maintenance organization P-value - probability value, < 0.05 = Significant difference between the populations undergoing ST vs. MT

Characteristics	MT	ST	Total	p-value
Overall	1705	695	2400	
Age in years (%)				<0.0001
18-34	2.64	5.04	3.33	
35-49	12.61	21.58	15.21	
50-64	36.07	35.97	36.04	
>=65	48.68	37.41	45.42	
Gender (%)				0.9438
Male	53.08	53.24	53.12	
Female	46.92	46.76	46.88	
Race (%)				0.7186
White	68.62	69.78	68.96	
Black	17.89	17.27	17.71	
Hispanic	4.99	5.04	5	
Others	2.35	4.32	2.92	
Missing	6.16	3.6	5.42	
Comorbidities (%)				
Obesity	34.6	40.29	36.25	0.0086
Hypertension	65.1	55.4	62.29	<0.0001
Diabetes mellitus with chronic complications	12.32	17.99	13.96	0.0003
Diabetes mellitus without chronic complications	14.66	6.47	12.29	<0.0001
Congestive heart failure	20.82	43.17	27.29	<0.0001
Valvular heart disease	7.62	16.55	10.21	<0.0001
History of chronic pulmonary disease	18.48	10.79	16.25	<0.0001
Peripheral vascular disease	6.45	7.19	6.67	0.5083
Coagulopathy	22.87	41.73	28.33	<0.0001
Solid tumor without metastasis	7.04	5.04	6.46	0.0703
Metastatic cancer	7.04	2.88	5.83	< .00001
Liver disorders	4.11	5.04	4.37	0.3121
Weight loss	3.81	7.91	5	<0.0001
Alcoholism	3.52	6.47	4.38	0.0013
Neurological disorders	7.62	12.95	9.17	<0.0001
Renal failure	8.8	8.63	8.75	0.897
Hypothyroidism	12.32	11.51	12.08	0.5827
Anemia deficiency	24.63	15.11	21.87	<0.0001
Fluid and electrolyte disorders	38.71	61.15	45.21	<0.0001
Depression	11.73	10.79	11.46	0.5125
Elixhauser comorbidity scale				<0.0001
1	4.99	6.47	5.42	
2	8.21	15.11	10.21	
3	86.8	78.42	84.38	
Median household income (%)				0.0004
1st quartile	27.86	25.18	27.08	
2nd quartile	29.03	23.74	27.5	
3rd quartile	25.22	25.9	25.42	
4th quartile	17.01	23.74	18.96	
Primary Insurance (%)				0.3478
Medicare/Medicaid	56.3	53.96	55.62	
Private including HMO	37.83	39.57	38.33	
Uninsured/Self-pay	5.28	6.47	5.63	
Hospital bed size (%)				<0.0001
Small	12.9	7.19	11.25	
Medium	24.63	15.11	21.88	
Large	62.46	77.7	66.87	
Hospital type (%)				0.0489
Rural	2.64	0.72	2.08	
Urban-nonteaching	15.84	14.39	15.42	
Teaching	81.52	84.89	82.5	
Hospital region (%)				<0.0001
Northeast	11.44	20.86	14.17	
Midwest	26.69	20.14	24.79	
South	40.76	47.48	42.71	
West	21.11	11.51	18.33	
Day of admission				0.004
Weekday	79.47	74.1	77.92	
Weekend	20.53	25.9	22.08	
Source of admission (%)				<0.0001
Transfer from another hospital or another health facility	25.81	46.76	31.87	
Emergency department	74.19	53.24	68.13	
Type of admission (%)				0.7966
Emergent or urgent	94.71	94.96	94.78	
Elective	5.29	5.04	5.22	

In-hospital outcomes

The overall unadjusted in-hospital mortality (IHM) for patients diagnosed with saddle PE undergoing ST was 15.1%, whereas 35.3% of the ST-treated study population was discharged to a skilled nursing facility (DTF) with an overall stay of 13.2 days. The overall unadjusted IHM for patients diagnosed with saddle PE undergoing MT was 11.1%, and 23.5% of the MT-treated study population was DTF, with an overall length of stay of seven days. In summary, MT had significantly lower mortality, need for postoperative nursing facilities, and length of stay (p<0.0001). Following ST, the most common complications were congestive heart failure (CHF) at 43.2% and atrial fibrillation (AF) at 28.8%. After MT, the most common complications were vascular events (24.1%) and CHF (20.8%). ST had a significantly higher risk of CHF and AF (p<0.0001), while MT had a significantly higher risk of vascular events and MI (p<0.0001). Extracorporeal membrane oxygenation (ECMO) was used on 1.4% of the ST patients and 0.9% of the MT patients (p<0.0001). 33.8% of patients that underwent ST were on intermittent mechanical ventilation (IMV), while only 17.6% of the MT patients required IMV (p<0.0001). Table 2 describes post-procedural outcomes of MT and ST.

**Table 2:** Outcomes after intervention (ST or MT) MT – mechanical thrombectomy; ST – surgical thrombectomy; DTF – discharge to a skilled nursing facility; LOS – length of stay; IMV – intermittent mechanical ventilation; ICeH – intra-cerebral hemorrhage; MI – myocardial infarction; CHF – congestive heart failure

	MT	ST	p-value
Overall	1705	695	
In-Hospital Mortality	11.1	15.1	<0.0001
DTF	23.5	35.3	<0.0001
LOS (days)	7.0	13.2	<0.0001
IMV	17.6	33.8	<0.0001
ICeH	0.6	0.7	<0.0001
Vascular event	24.1	22.3	<0.0001
MI	8.5	4.3	<0.0001
CHF	20.8	43.2	<0.0001
Atrial fibrillation	13.8	28.8	<0.0001

## Discussion

Temporal trends of surgical and mechanical thrombectomy

There has been a steady increase in MT-capable centers in the United States over the past 10 years, more commonly around major cities [15]. A study from 2019 demonstrated a 10-fold increase in the utilization of catheter-directed thrombolysis (CDT) over 12 years [16,17], while an earlier study from 2012 reported a six-fold increase over five years [18]. The increase in mechanical thrombectomy utilization results from increased access to capable centers and the decreased overall morbidity and mortality associated with the intervention. In 2014, the FDA approved using mechanical thrombectomy to treat PE [19]. The use of MT for the treatment of PE has been increasing since then, and our data reflected the same trend. On the other hand, the use of surgical thrombectomy has remained relatively stable over the years. This was expected, as there have been no innovations or changes to specific indications for its use [17].

Baseline characteristics of the ST vs. MT groups

Most of our study population fell into the >50 years age group. This matches the tendency of pulmonary embolism to occur in elderly patients more than in younger ones [4,20]. On closer examination, the >65 years age group primarily underwent MT, while the 18-34- and 35-49-year age groups mainly underwent ST. Because of the high surgical risk of ST in the elderly, physicians prefer catheter-based thrombectomy or thrombus fragmentation over surgery [21]. The majority of our study population is also White - not surprising, as the population of the United States is predominantly White.

Patients with hypertension, chronic pulmonary disease, and anemia were more likely to undergo MT over ST. This was an expected finding, as these comorbidities are associated with more significant risks from anesthesia and blood loss during surgery. On the other hand, patients with obesity, congestive heart failure (CHF), and valvular heart disease (VHD) were more likely to undergo ST. Existing literature has no explanation for this. The only related studies we could find reported patients developing chronic pulmonary hypertension after treatment of PE and requiring ST as treatment [22,23]. We hypothesize that the increased pulmonary pressures associated with obesity, CHF, and VHD make it harder to proceed with mechanical thrombectomy.

Patients with complications from diabetes mellitus tended to undergo ST, while those with no complications underwent MT. Previous studies have demonstrated poor outcomes after MT in diabetics but were unable to determine the causal relationship between hyperglycemia and poor prognosis [24]. Another study determined that the lacking collateral circulation in diabetics was a significant contributor to the worse results in diabetics after MT [25].

Patients with metastatic cancer were also more likely to undergo MT. This is likely because metastatic cancer increases surgical risks, and an unnecessarily invasive procedure in a patient with a limited lifespan is relatively contraindicated [21,26]. The focus is instead on minimizing complications after the intervention to maximize the quality of life.

Surprisingly, patients with coagulopathy were more likely to undergo ST over MT. We expected the opposite, as the patients would be more prone to intra-operative and postoperative bleeding. We could not find any literature explaining this phenomenon. Still, we hypothesize that patients with coagulopathy are either at increased risk for the formation of additional thrombi or progression to DIC and thus require more drastic intervention.

Patients admitted on weekends were more likely to undergo ST, while those admitted on weekdays were more likely to undergo MT. This is probably due to the availability of personnel. MT requires skilled interventional radiologists and the staff to operate the specialized equipment. They are less likely to be available on weekends. On the other hand, most hospitals have surgeons and OR staff available for emergencies over the weekend. Given that massive saddle PE is an acute emergency requiring immediate treatment [27], it is only logical that they proceed with ST when MT is unavailable.

On a similar note, patients transferred from other facilities were more likely to undergo ST, while those admitted directly from the emergency department of the hospital were more likely to undergo MT. The reasoning here is two-fold-patients transferred from other locations are likely to have more severe forms of the disease and have more time to deteriorate during transit. They tend to be unstable and require emergent surgery. In comparison, patients admitted directly from the emergency department are likely to receive treatment faster, allowing them to undergo MT [28].

In-hospital outcomes of ST and MT

Saddle PE is an acute, life-threatening condition associated with elevated right atrial pressure, profound hypoxemia, and heart failure, even when treated promptly, with a high mortality rate. Our study reported the same for both the ST and MT groups, with significantly higher mortality in the ST group (15.1% vs. 11.1% for ST and MT, respectively). Similar findings have been reported in other studies [29]. The higher mortality of ST is also expected and matches prior results. Unstable, critically ill patients tend to undergo rescue surgery (ST), and the inherent risks and complications of surgery add to the mortality rate in the ST group [20]. For the same reasons, patients undergoing ST tend to have more extended hospital stays and a higher chance of being discharged to a skilled nursing facility instead of going home after their postoperative stay [30].

Congestive heart failure was the most common periprocedural complication reported in both groups, with a significantly higher occurrence in the ST group. This finding has been reported in prior studies [31] and has multiple contributing factors. One, it is a consequence of cardiopulmonary bypass performed as part of the ST procedure [30]. Two, as discussed before, critically ill patients and severely hemodynamically compromised patients are likely to undergo ST [32]. And three, patients requiring ST generally have a greater risk of right ventricular strain and myocardial infarction, contributing to the development of heart failure. However, despite the high incidence of CHF, various studies have demonstrated excellent cardiac recovery after both procedures.

We also found that new-onset atrial fibrillation was a frequent adverse event after both procedures, more commonly following ST than MT. PE itself may be the trigger for AF through increased right atrial pressure and subsequent right atrial strain-with ST patients having a higher risk than MT patients, as they tend to have more severe obstructions and right heart strain (as discussed earlier) [33]. There is relatively little data available on this topic, and the incidence of postoperative atrial fibrillation may be underestimated [34].

Vascular events were another significant periprocedural complication for both MT and ST, with MT having a small (1.8%) but significantly higher incidence. Patients undergoing ST usually require CPB, which exposes them to systemic anticoagulation. This increases the risk of widespread bleeding, including intracranial hemorrhage. In contrast, the MT group has a higher proportion of patients who failed or have absolute contraindications to systemic thrombolysis and high surgical risk-i.e., patients who already have an increased risk of bleeding complications [35-37].

33.8% of ST patients and 17.6% of MT patients required intermittent ventilation (IMV) during or after the procedure (p < 0.001). The hypoxic condition generated by saddle PE is one of the main contributors to the initiation of IMV, after which the patients are maintained on artificial ventilation until the saddle PE is resolved [38,39]. Patients requiring ST are usually more critically ill and frequently have been previously mechanically ventilated, increasing the chances that they may need IMV [40]. Also, ST is often used as a last resort for patients who failed to respond or have contraindications to initial management and have contraindications to MT-i.e., patients with a higher risk of needing IMV [6,41].

Limitations

This study is based on a retrospective analysis of NIS data. This analysis is inherently vulnerable to the effects of missing data or administrative errors in data entry. As ICD-9 and ICD-10 diagnostic codes were used to extract data, errors in coding the diagnosis or description of saddle PE can also be assumed. However, the size of the dataset used will offset the effect of these errors. Also, as NIS is a discharge database, each hospitalization is unique but the same patient may be recorded multiple times for multiple admissions, inflating the study population. For the same reason, it is impossible to establish a temporal relationship between the intervention (ST or MT) and the outcomes or complications. Nor can we identify whether the complications (such as AF or CHF) predated the admission for saddle PE. Our data was also limited to the three-year period from 2016 to 2018, as mechanical thrombectomy for treatment of PE was only approved in 2014. This relatively short study period is another limitation of our study.

## Conclusions

The use of mechanical thrombectomy has been steadily increasing in the study period, while surgical thrombectomy has held steady. Surgical thrombectomy is more common in large/teaching hospitals, weekend admissions, and patients transferred from other facilities, while mechanical thrombectomy is more common in the elderly (age >65 years) and patients with a higher comorbidity score. Mechanical thrombectomy is also associated with decreased mortality and length of hospital stay compared to surgical thrombectomy and has a lower risk of complications.
